# Artificial intelligence assisted clinical fluorescence imaging achieves in vivo cellular resolution comparable to adaptive optics ophthalmoscopy

**DOI:** 10.1038/s43856-025-00803-z

**Published:** 2025-04-23

**Authors:** Joanne Li, Jianfei Liu, Vineeta Das, Hong Le, Nancy Aguilera, Andrew J. Bower, John P. Giannini, Rongwen Lu, Sarah Abouassali, Emily Y. Chew, Brian P. Brooks, Wadih M. Zein, Laryssa A. Huryn, Andrei Volkov, Tao Liu, Johnny Tam

**Affiliations:** National Eye Institute, National Institutes of Health, Bethesda, MD 20892 USA

**Keywords:** Imaging, Eye diseases

## Abstract

**Background:**

Advancements in biomedical optical imaging have enabled researchers to achieve cellular-level imaging in the living human body. However, research-grade technology is not always widely available in routine clinical practice. In this paper, we incorporated artificial intelligence (AI) with standard clinical imaging to successfully obtain images of the retinal pigment epithelial (RPE) cells in living human eyes.

**Methods:**

Following intravenous injection of indocyanine green (ICG) dye, subjects were imaged by both conventional instruments and adaptive optics (AO) ophthalmoscopy. To improve the visibility of RPE cells in conventional ICG images, we demonstrate both a hardware approach using a custom lens add-on and an AI-based approach using a stratified cycleGAN network.

**Results:**

We observe similar fluorescent mosaic patterns arising from labeled RPE cells on both conventional and AO images, suggesting that cellular-level imaging of RPE may be obtainable using conventional imaging, albeit at lower resolution. Results show that higher resolution ICG RPE images of both healthy and diseased eyes can be obtained from conventional images using AI with a potential 220-fold improvement in time.

**Conclusions:**

The application of using AI as an add-on module for existing instrumentation is an important step towards routine screening and detection of disease at earlier stages.

## Plain language summary

Advanced imaging methods that allow single cells to be seen in the living human eyes are not always available to ophthalmologists working in the clinic. We combined artificial intelligence (AI) with standard clinical imaging to visualize cells within the retina, a part of the eye, that are critical for maintaining vision and having healthy eyes. We found the cells could be seen using a commonly used fluorescent dye and standard clinical imaging equipment augmented with AI. The resulting AI images are comparable to those acquired using advanced imaging technology. By making cellular-level information more readily available in routine clinical practice, it may be possible to detect diseases earlier.

## Abstract (web summary)

Li et al. use standard clinical imaging of living human eyes and apply artificial intelligence (AI) to obtain cellular information. The improvements in resolution possible with indocyanine green images obtained using adaptive optics can also be achieved using AI, with a 220-fold improvement in throughput.

## Introduction

Advances in biomedical optics research have led to the ability to image at the cellular scale in the living human body. However, while most clinical imaging instruments are able to visualize tissue-level details, it remains challenging to deploy more sophisticated instrumentation capable of resolving individual cells for routine clinical use. This challenge is due in part to the need for specialized expertise for implementing, operating, and maintaining such instrumentation. Furthermore, as technological development accelerates, there is a growing gap between the current imaging instrumentation used in clinical practice and the next generation of advanced imaging that is generally only available in research settings with dedicated technical support staff. Increasingly, artificial intelligence (AI) is being applied to ophthalmology^1–3^. In this paper, we further extend the application of AI by introducing stratified cycleGAN, an AI network as a virtual image enhancement module that can be added on to existing clinical equipment. The AI network described in this paper enables high-end images to be obtained using only standard clinical imaging approaches.

AI has already revolutionized the field of medical imaging by providing tools for disease detection and image analysis^4–7^. In particular, deep learning has been increasingly implemented into various medical imaging analyses, such as segmentation and disease prediction^6^. Recent studies have shown that the deep learning approach can be applied to image denoising and image reconstruction^4,8,9^. Specifically, methods designed for style transfer have become widely-utilized for medical image generation in both radiology and ophthalmology^10–13^. Examples include estimating high-quality full-dose computed tomography^8^ or position emission tomography^14^, increasing the resolution of magnetic resonance images from lower resolution images^15^, and reducing speckle noise in optical coherence tomography images^13,16^.

Ophthalmic imaging provides a glimpse into the cells of the central nervous system, including the retinal pigment epithelial (RPE) cells. Intravenously-injected indocyanine green (ICG) dye has been shown to be valuable for visualizing deeper tissue layers due to its near-infrared emission spectrum^17^. Recently, ICG dye^18–22^ has been introduced as a method to label RPE cells. This approach is particularly valuable for monitoring diseases where RPE cells are thought to have a central role in the initiation and progression of vision loss, including age-related macular degeneration (AMD), retinitis pigmentosa, vitelliform macular dystrophy, choroideremia, and Stargardt disease^23–27^. Visualization of cells labeled by ICG has thus far been primarily carried out using adaptive optics (AO) imaging^19,25,28^, except in diseases such as choroideremia, where the cells are enlarged to a greater extent and are visible even using conventional imaging^23^. Even though AO imaging provides exquisite detail and achieves cellular level resolution^29–31^, it remains a highly-specialized technique not widely accessible in most eye clinics.

Given that RPE cells are at or near the limit of resolution achievable using standard clinical imaging (~10–15 µm), we explored the possibility of visualizing individual RPE cells without the use of AO by leveraging AI. We show that images obtained using clinical imaging instrumentation can be enhanced using AI to a level comparable to those obtained using high-end, research-grade AO imaging. The reported results introduce an AI-based strategy to virtualize AO and thereby circumvent technological barriers that would have otherwise hindered the adoption of new technology. In particular, we demonstrate the potential benefit of AI for ushering in a new era of imaging – AI-assisted imaging, which can serve as a transformative step towards the routine assessment of tissue health at the cellular level in clinical practice.

## Methods

### Subjects

Healthy eyes from 22 individuals from previously collected data were included in this study (Supplementary Table 1). In addition, four eyes from patients with age-related macular degeneration (AMD), vitelliform macular dystrophy, retinitis pigmentosa, and choroideremia (female carrier) were included (Supplementary Table 2). Both subject recruitment and data collection were conducted at the National Eye Institute. Prior to imaging, written, informed consent was obtained from all subjects after the nature of the research and possible consequences of the study were explained. In addition, a dilated fundus examination was conducted on all participating subjects to determine eligibility (healthy vs. diseased eyes). Ethnicity was self-reported by the participants. This study was approved by the institutional review board of the National Institutes of Health and was conducted in accordance with the Declaration of Helsinki (NCT02317328; https://clinicaltrials.gov).

### Multimodal imaging

#### AO-enhanced ICG (AO-ICG) imaging

ICG was administered through intravenous injection at a dose of 25 mg in 3 mL according to the standard of care at the National Eye Institute. AO imaging was performed using a custom-built multimodal AO imaging system^18,19^. Approximately one hour after ICG injection, AO imaging was acquired from the central macula to approximately 5 mm away along the temporal direction. An overview of ICG imaging of retinal pigment epithelial (RPE) cells is shown in Supplementary Fig. 1. The total AO imaging time (including time for breaks) was approximately 40 min to one hour per eye. A computer controlled fixation system was used^32^ and subjects were encouraged to blink naturally and take frequent breaks.

The custom-built AO instrument simultaneously acquires confocal reflectance and AO-ICG images to visualize photoreceptors^33^ and ICG-labeled RPE cells^18^, respectively. The system uses a 790 nm superluminescent diode (SLD) (S-790-G-I-15-M, Superlum, Ireland) for imaging and an 880 nm SLD (SLD-mCS-341-HP1-SM-880, Superlum, Ireland) for wavefront sensing in order to correct for ocular aberrations using AO. The light levels measured at the cornea were maintained below 135 µW for the 790 nm light source and under 40 µW for the 880 nm light source, which were both below the maximum permissible exposure limits set by the American National Standards Institute standard Z80.36-2021^34^.

#### Conventional ICG imaging

Conventional imaging was conducted using a commercially-available scanning laser ophthalmoscope (Spectralis, Heidelberg Engineering). One-hundred-frame averaged 30° field of view (FOV) Automated Real-time Tracking (ART) images of the retina were obtained in high resolution mode at least 45 min after ICG administration.

#### High magnification mode (HMM) ICG imaging

An add-on lens (high magnification module, HMM, Heidelberg Engineering) was attached to the Spectralis scanning laser ophthalmoscope (Heidelberg Engineering). The focus value was set to a similar value used for conventional ICG imaging, then averaged ART (100 frames) images were acquired in high resolution mode at locations matching the retinal region imaged by AO-ICG and conventional ICG imaging.

### Image analysis

#### AO images

For AO images, eye motion was corrected through strip-based image registration based on simultaneously collected photoreceptor images acquired by AO confocal reflectance imaging^35^, and then averaged AO-ICG images were assembled into a larger montage based on retinal features in overlapping areas.

To quantify RPE across eccentricities, regions of interest (ROIs) (approximately 150 × 150 µm^2^) were selected from each AO-ICG montage starting from the fovea and extending out to 5 mm in the temporal direction with 0.5 mm increments. The fovea was determined based on the average of one or more videos acquired to capture the preferred retinal locus (PRL) of fixation, during which the subject is asked to fixate at the center of the imaging raster. In all cases, the location of the fovea (based on PRL) was consistent with the location of the foveal contour as seen using optical coherence tomography. A total of 190 ROIs from 26 eyes were selected using the PRL as the origin for calculating retinal eccentricity. Scaling of image distances from degrees to millimeters was calculated based on a modified Bennett-Rabbett model eye using the ocular biometry measurements (axial length, corneal curvature, and anterior chamber depth) acquired after dilation (IOL Master, Carl Zeiss Meditec)^36^. Identification of RPE cells was performed on ROIs using a previously published detection software^37^ followed by manual correction. This algorithm uses a regression-based probability map to determine the center points of ICG labeled RPE cells. Following this initial seeding, the identified cell centers in all images were sequentially evaluated and iteratively adjusted by at least two expert graders until full consensus between graders was achieved. The identified cell centers were used to construct a Voronoi diagram for visualization purposes. RPE density was calculated after discarding incomplete cells at the boundaries of the images^25^ and RPE spacing was calculated based on the density recovery profile^38^.

Averaged RPE spacing and density values were calculated across all subjects at each eccentricity. To visualize relationships between changes in RPE spacing/density and age/sex, measurements were normalized by dividing each measurement by the expected average value corresponding to each eccentricity^39^. The number of neighboring cells was calculated based on the RPE detection result. After cells are detected in each ROI image, detected cells at the image boundaries of ROI were discarded. Using the remaining cells (not at image boundaries), the number of neighboring cells was calculated for each RPE cell.

#### Conventional ICG images

No additional post processing was performed to the conventional ICG images. The exported images were scaled appropriately to spatially register conventional ICG images to AO-ICG montages. RPE quantification was performed using the same method as performed for AO images. The contrast of conventional ICG images shown in the manuscript was enhanced for visualization purposes only.

#### HMM ICG images

No additional post processing was performed to the HMM images. The exported images were scaled appropriately to spatially register HMM ICG images to AO-ICG montages. RPE quantification was performed using the same method as performed for AO images. The contrast of the HMM ICG images shown in the manuscript was enhanced for visualization purposes only.

### AO and non-AO comparison

Six eyes had AO-ICG, conventional ICG, and HMM images and were used for AO and non-AO comparison. For each eye, AO-ICG montages were spatially co-registered to the corresponding conventional and HMM images based on matching retinal features. ROIs in conventional and HMM images across eccentricities were obtained at the same retinal locations as AO-ICG ROIs, and then RPE cells in each image were analyzed using the same procedures as performed for AO images.

### AI image enhancement

Enhancement of conventional images was performed using stratified cycleGAN^10^. Briefly, a total of 1430 pairs of AO and conventional images were collected from 10 healthy human subjects (28.8 ± 8.6 years, Supplementary Table 1). Registered pairs of AO and conventional ROIs were selected from a larger montage constructed from overlapping images, avoiding any areas with montaging artifacts such as incomplete overlap between images or image distortion due to eye motion. Eighty percent of the images were used as training data and the remaining 20% were reserved for testing. The training process consists of two steps (Supplementary Fig. 2). The first step involves assigning numerical image quality grades (0 = poor, 1 = moderate, 2 = good) to AO images (high-quality images, HQ) using semi-supervised pseudo-labeling^40^, which is a semi-supervised learning technique to train the AI model to classify poor, moderate, and good images. Briefly, a small set of ground truth AO images (HQ) were manually assigned grading scores (0, 1, 2). These images were then used to train a classification model (C) to predict the grading scores for the remaining unlabeled HQ images. In the second step, the corresponding conventional low quality (LQ) images and the image quality grades are sent to a generator (G1) to generate high-quality images (HQ_g_). The generated HQ_g_ images are examined by a discriminator (D1) by comparing it against the ground truth (AO images, HQ) and providing an image validity result and an image quality grade. The quality of the generated HQ_g_ images is also checked by going through a second generator-discriminator (G2-D2) pair to produce generated low-quality images (LQ_g_) and be compared against the original input conventional images (LQ) using generation and cycle consistency loss functions. In this training model, both generators (G1 and G2) start with stride-2 convolution, followed by 9 residual blocks, and two fractionally-strided convolutions with the input being 256×256-pixel images. The data input for G1 also includes the grading score c that is converted into a 256×256 image. The discriminator (D1 and D2) networks use PatchGANs, in which D1 also has a connected layer to perform the image grading (0, 1, 2). Together, the additional image details gained from this iterative process ensure the reliable generation of HQ_g_ images. Validation of the AI model was performed based on objective cell detection results on the generated AI-ICG images compared to the results from the AO-ICG images (ground truth). The objective cell detection accuracy was assessed using an automated cell detection algorithm^10,37^.

Selected conventional images from healthy eyes that were not included in the training or testing data were used to evaluate the effectiveness of the enhancement software. Pairs of AO and conventional images from identical retinal locations were first spatially aligned based on ICG mosaic pattern (AO served as ground truth). Conventional images were then cropped to smaller ROIs (~250 × 250 µm) and processed by the enhancement software to generate AI-ICG images.

The sharpness of the image features in AI-ICG and conventional ICG images was calculated to quantitatively demonstrate an improvement in image quality after image enhancement. Sharpness was estimated using the gradient magnitude, calculated by dividing the sum of all gradient norms by the number of available pixels^41^. After the sharpness of each image was calculated, the values were normalized to the AO-ICG images to facilitate comparisons.

### Statistical analysis

The relationship between RPE spacing/density and sex/age was analyzed using a two-tailed *t* test, and changes in RPE spacing/density across different ethnicities was evaluated by Kruskal-Wallis paired with Tukey’s honest significant difference test. For the AO and non-AO comparison, Kruskal-Wallis combined with Tukey’s honest significant difference test was performed for the spacing/density measurements from six eyes to evaluate significant differences across the three sets of measurements. The magnitude of improvement in image feature sharpness after AI-assisted enhancement compared to the original conventional ICG images was analyzed using a two-tailed *t* test. *P* values under 0.05 are considered significant.

### Reporting summary

Further information on research design is available in the Nature Portfolio Reporting Summary linked to this article.

## Results

### Eccentricity, sex and age dependent changes in RPE revealed using adaptive optics

**Fig. 1:**
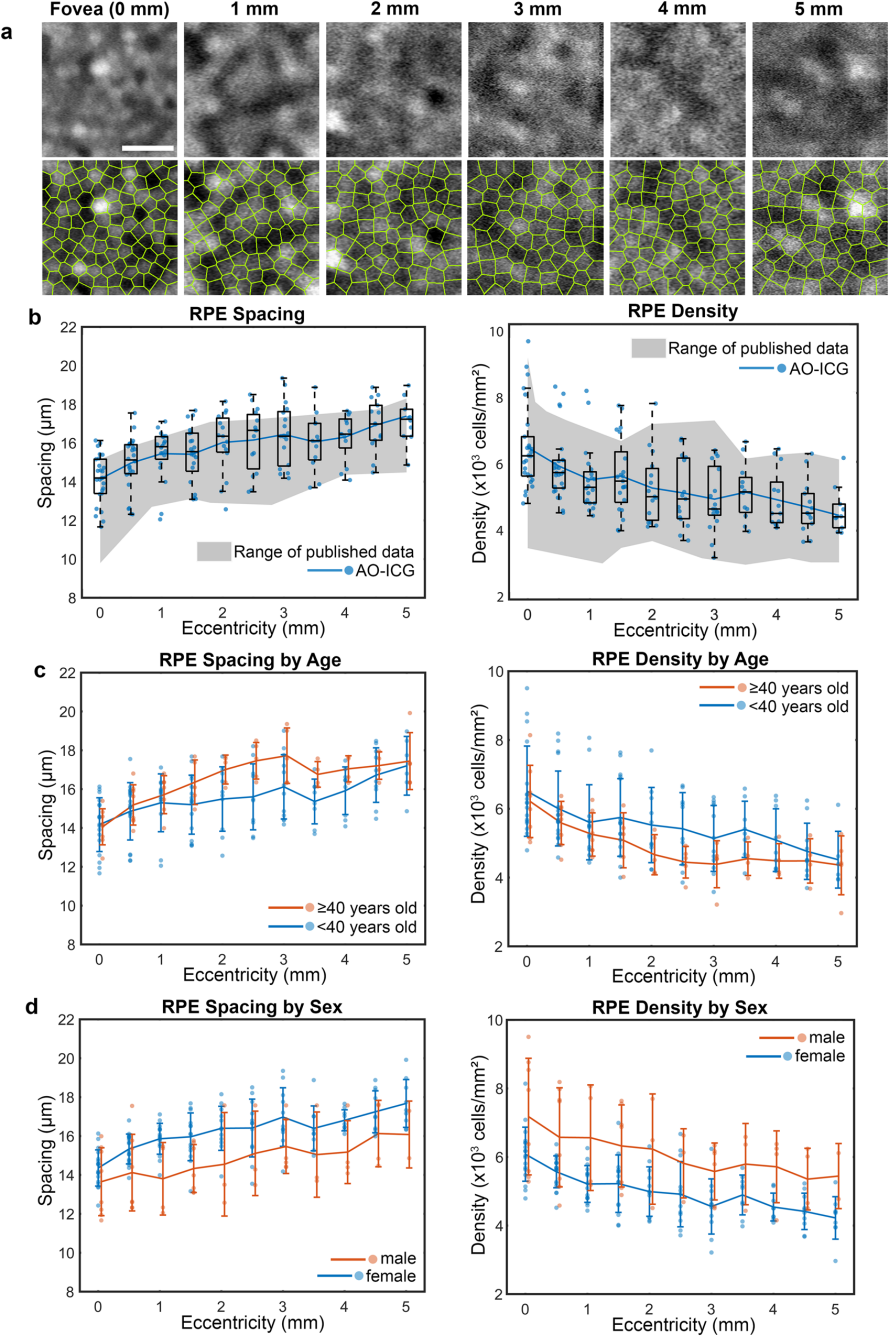
Late phase adaptive optics enhanced indocyanine green (AO-ICG) imaging enables measurement of retinal pigment epithelial (RPE) cell-to-cell spacing and density across the temporal eccentricities and age-related changes. **a** Example AO-ICG RPE mosaic at different eccentricities (top row) showing each RPE cell uniformly labeled by ICG. The corresponding Voronoi maps of the RPE cells (bottom row) are shown for visualization purposes. **b** RPE spacing and density measurements using AO-ICG images compared to previously published in vivo and ex vivo data (gray shaded area: range of previously reported values). Box plot: center line: median, box limits: upper/lower quartiles, whiskers: 1.5x interquartile range; blue markers: individual data points calculated from each location in each eye (*n* = 190 locations in this plot); blue line: averaged measurements across all eyes based on AO-ICG. **c** RPE spacing and density comparison between <40 (*n* = 130 locations) and ≥40 (*n* = 60 locations) years old. In general, RPE spacing is larger in ≥40-year-old cohort at most eccentricities in this study. RPE density is smaller at most eccentricities in the ≥40-year-old cohort. Line: mean ± SD; markers: individual data points. **d** RPE spacing and density comparison between male (*n* = 52 locations) and female (*n* = 138 locations). At all eccentricities, females have higher RPE spacing and lower density. Line: mean ± SD; markers: individual data points. Scale bar: 50 µm.

Since 2016^18^, the application of using ICG for quantifying RPE cells has been largely restricted to AO instrumentation, called AO-enhanced ICG (AO-ICG). Although RPE cells are also visible in early phase of AO-ICG^20^, in this paper we focus on late phase AO-ICG, approximately 45 min after intravenous injection of ICG dye. At this time point, a hexagonal pattern of ICG fluorescence can be consistently observed in the RPE cells, with neighboring cells exhibiting different levels of ICG intensity (Supplementary Fig. 1)^19,21,28^. The origin of the ICG signal has been confirmed previously in mouse eyes^18,19,23,42,43^, and by other AO modalities, including infrared-autofluorescence, darkfield, and optical coherence tomography (Supplementary Fig. 3)^18,28^. In order to establish how the information contained within AO-ICG images might be clinically relevant, a retrospective analysis was performed on 26 healthy eyes from 20 individuals across different ages (33.6 ± 13.8 years [mean ± SD], range 22–63; see Supplementary Table 1), which revealed an eccentricity-dependent change in cell-to-cell spacing and density, consistent with but expanding upon previous reports (Fig. 1). As has been previously reported^19^, the heterogeneous ICG fluorescence pattern was robustly observed across 100% of the subject cohort, which fortuitously enables neighboring RPE cells to be distinguished from each other.

Based on our previously established late phase ICG imaging technique and criteria for identifying RPE cells^37^ (Supplementary Fig. 1), RPE cell-to-cell spacing and density measurements at each eccentricity show that RPE spacing is the lowest at the fovea (14.1 ± 1.2 µm, mean ± SD), gradually increasing to 17.4 ± 1.4 µm at an eccentricity of 5 mm (Fig. 1b). In contrast, the RPE density is highest at the fovea (6419 ± 1224 cells/mm^2^), decreasing to 4444 ± 801 cells/mm^2^ at an eccentricity of 5 mm in the temporal direction (Fig. 1b). These trends are consistent with in vivo and ex vivo published data^18,19,44–57^ (gray shaded area in Fig. 1b: range of previously reported values). In addition, the RPE cell detection result shows that most detected RPE cells have 6 neighboring cells (5.98 ± 0.03, mean ± SD). This observation is also consistent with previous reports^44^.

There were also differences in RPE cell parameters due to age. Normalized RPE spacing and density (normalized to average expected values for each eccentricity) revealed that RPE spacing is significantly larger and density is significantly smaller in ≥40-year-old cohort (Supplementary Fig. 4, *p* = 1 × 10^–4^ [spacing], *p* = 2 × 10^–4^ [density], two-tailed *t* test). In particular, RPE spacing appears to become larger while the density measurements become smaller with age across most retinal eccentricities (Fig. 1c). This observation builds upon previous findings of RPE density decreasing with age as reported in both histology^45,47,48,58^ as well as in vivo human imaging^59^.

Additionally, comparison of RPE spacing between females and males suggested that RPE spacing is larger and density is smaller at all eccentricities in female eyes (Fig. 1d). RPE spacing values in female eyes is on average 8% larger than in male eyes (*p* = 2 × 10^–10^, two-tailed *t* test); correspondingly, RPE density in female eyes is on average 21% lower than in male eyes (*p* = 1 × 10^–13^, two-tailed *t* test) as shown in Supplementary Fig. 4. These differences did not appear to arise due to differences in axial length (female: 23.7 ± 0.9, male: 23.8 ± 0.8 mm, *p* = 0.73) or in age (female: 36.3 ± 13.7, male: 30.4 ± 13.4, *p* = 0.32). Intriguingly, the slightly larger RPE cell spacing observed in female eyes is similar to the slightly larger cone photoreceptor diameters observed in female subjects as has been previously reported in a separate subject cohort^60^. There were no apparent differences in the RPE across ethnicity (Supplementary Fig. 4).

Taken together, these results indicate that the heterogeneous pattern of RPE fluorescence established by intravenous ICG injection can be robustly imaged using AO (AO-ICG) for the purposes of obtaining cellular information of RPE in living human eyes. However, whether this same information can be gleaned using more routine clinical imaging instrumentation remains to be demonstrated.

### Visualizing RPE cells using standard clinical scanning laser ophthalmoscopy

Although AO can provide cellular details of RPE cells due to the improved lateral resolution compared to the current standard clinical ophthalmic imaging, AO technology is not readily accessible in most eye clinics. However, cellular-level information of RPE may be possible without AO since the size of the smallest RPE cells (~14 µm at the fovea) is near the reported lateral resolution (~10–15 µm) of some commercially-available clinical imaging systems, such as the scanning laser ophthalmoscope (SLO) (Spectralis, Heidelberg Engineering). Here, we sought to explore the use of a hardware-based approach to explore the feasibility of upgrading clinical imaging with the capability of cellular scale imaging.

A recently-introduced custom lens add-on (High Magnification Module, HMM, Heidelberg Engineering), originally developed to enable visualization of cone photoreceptors using a clinical instrument without the need for AO^61^, was instead used to obtain higher resolution images of the fluorescently labeled RPE cells approximately one hour after intravenous injection of ICG (late phase ICG) (*n* = 6 eyes imaged using both AO and HMM, Supplementary Table 1). Our results indicate that HMM imaging can also provide images of RPE cells that are comparable to AO-ICG images.

**Fig. 2:**
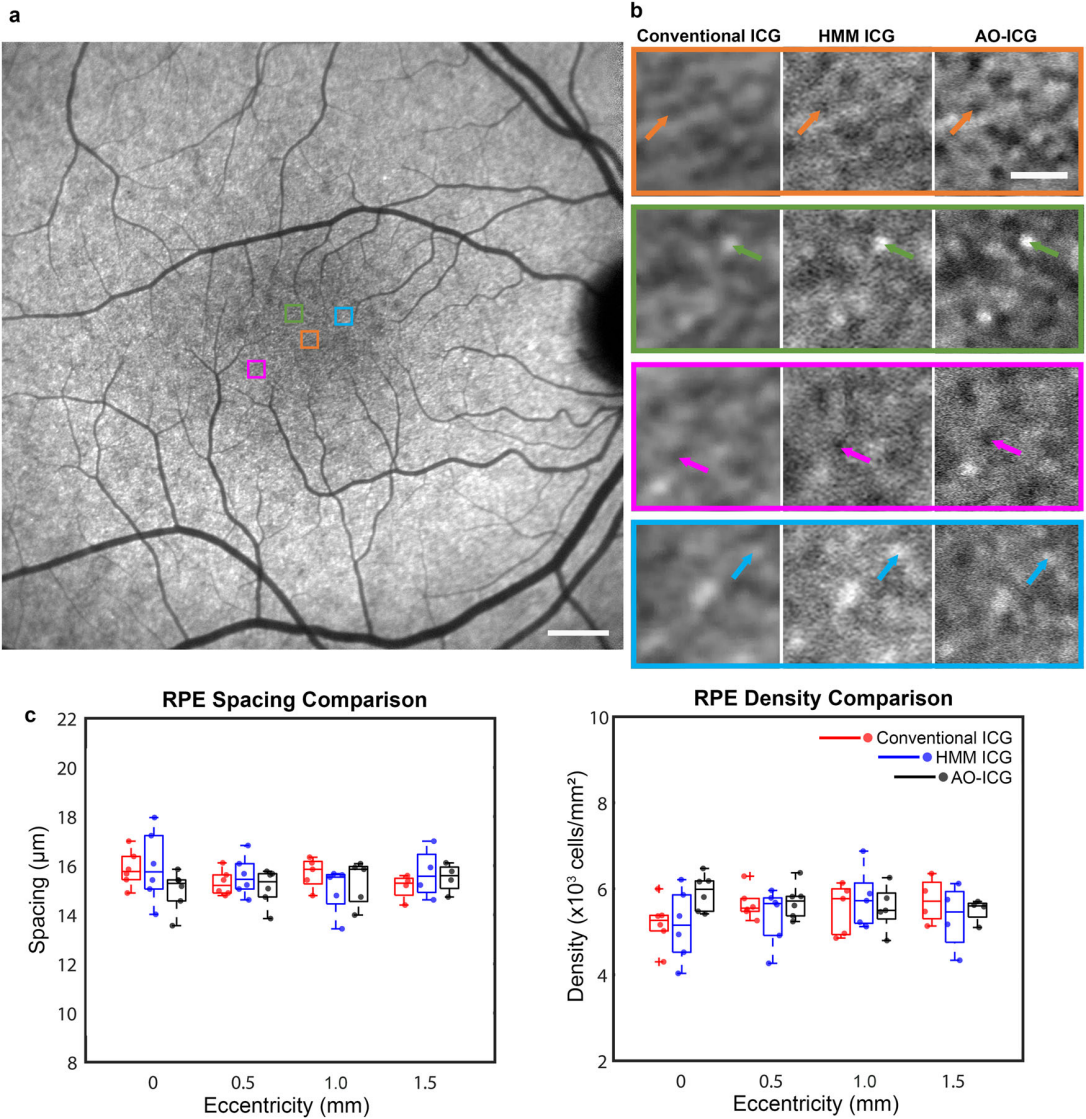
Late phase indocyanine green (ICG) retinal pigment epithelial (RPE) cell mosaic is visible using both adaptive optics (AO) and non-AO (conventional and high magnification module) instruments. **a** Conventional image of late phase ICG mosaic from a 42-year-old healthy eye. Selected locations for visual comparison of ICG mosaic acquired from conventional ICG, high magnification module (HMM) ICG, and AO-ICG are shown by colored boxes. **b** Selected regions of interest (color matched boxes in 2a and 2b) show a consistent ICG pattern observed at each location by both AO and non-AO instruments, but with lower resolution in the non-AO images (the contrast of the non-AO images in (**b**) were adjusted for visualization purposes). Example of RPE cells at each location is shown by the color-matched arrows. **c** Box plots showing RPE spacing and density measurements from six eyes at various eccentricities based on conventional ICG (red), HMM ICG (blue), and AO-ICG (black) images (colored dots are individual data points of corresponding modalities, *n* = 21 locations per modality). No significant difference was observed across the three modalities, suggesting RPE cellular level information can be acquired by both AO and non-AO instruments. An additional example comparing AO and non-AO instruments is provided in Supplementary Videos 1 and 2. Scale bars: (**a**) 1 mm, (**b**) 50 µm.

Multiple retinal regions were imaged to compare the ICG RPE mosaic using AO, HMM, and a commercially available SLO with a standard 30° imaging lens (thereby referred to as “conventional”) (Spectralis, Heidelberg Engineering). A detailed comparison of conventional, HMM, and AO imaging modes is listed in Supplementary Table 3. Close observation reveals that the same ICG mosaic pattern could be seen across all three imaging modalities, but with varying levels of resolution (Fig. 2 and Supplementary Videos 1 and 2). From a larger view on the conventional image (Fig. 2a), one can see a mostly uniform ICG signal across the retina with a slight hypocyanescent region within the macula. This is distinct from the 787-nm autofluorescence images showing increased autofluorescence in the central macula relative to surrounding areas as acquired using SLO (Supplementary Fig. 5). In addition, it is important to note that because the ICG fluorescence signal is much stronger than the 787-nm autofluorescence from the retina^18^, variability in autofluorescence is not expected to have a strong impact on the ICG fluorescence signal. Upon closer view on both non-AO (conventional and HMM) and AO images (Fig. 2b), the mosaic pattern of varying ICG signals across individual cells can be better observed. The fact that the same heterogeneous pattern of fluorescence was observed using both non-AO and AO instruments not only corroborates our claim that the same fluorescent signal is being detected by multiple systems, but also indicates the possibility for detecting cellular structure without the need for AO instrumentation. In particular, availability of AO-ICG images acquired from the same retinal locations help to serve as a higher resolution ground truth for improving the interpretability of the lower resolution non-AO images.

Despite the lower resolution in non-AO images, the majority of individual RPE cells were still identifiable not only in the HMM ICG image, but also in the conventional ICG image, which has even lower lateral resolution than HMM. To assess whether reliable cellular information can be obtained using non-AO ICG images, RPE cells were quantified on both HMM and conventional images at the same ROIs as where AO images were acquired. RPE cell-to-cell spacing and density measurements obtained using non-AO images were comparable to ground truth measurements independently obtained from AO across multiple retinal locations (eccentricities) (Fig. 2c). Kruskal-Wallis combined with Tukey’s honest significant difference test performed separately for both spacing and density on the three sets of measurement revealed no significant difference among the three modalities (AO vs. HMM: *p* = 0.68, AO vs. conventional: *p* = 0.77, HMM vs. conventional: *p* = 0.99). This observation reveals an unexpected finding that non-AO images can contain RPE cellular information content in the context of late phase ICG imaging, even though they appear to have lower contrast and clarity when compared to AO-ICG images.

### AI-assisted imaging achieves cellular-level resolution faithful to high end AO imaging

Having established that the heterogeneous ICG fluorescence pattern represents a signal originating from the RPE that can be revealed using both AO as well as non-AO imaging techniques, here, we sought to explore the possibility of using AI to generate AO-like images from conventional ICG images. Leveraging pairs of AO-ICG images with corresponding non-AO conventional ICG images from different retinal locations collected from 10 subjects, we trained the stratified cycleGAN model^10^ to convert conventional images to AO-like images, and then used the fully trained model to generate AI-enhanced ICG (AI-ICG) images from never-seen conventional ICG images. The workflow of a trained image enhancement platform and the architecture of the AI training model are detailed in “Methods” section as well as Supplementary Fig. 2.

**Fig. 3:**
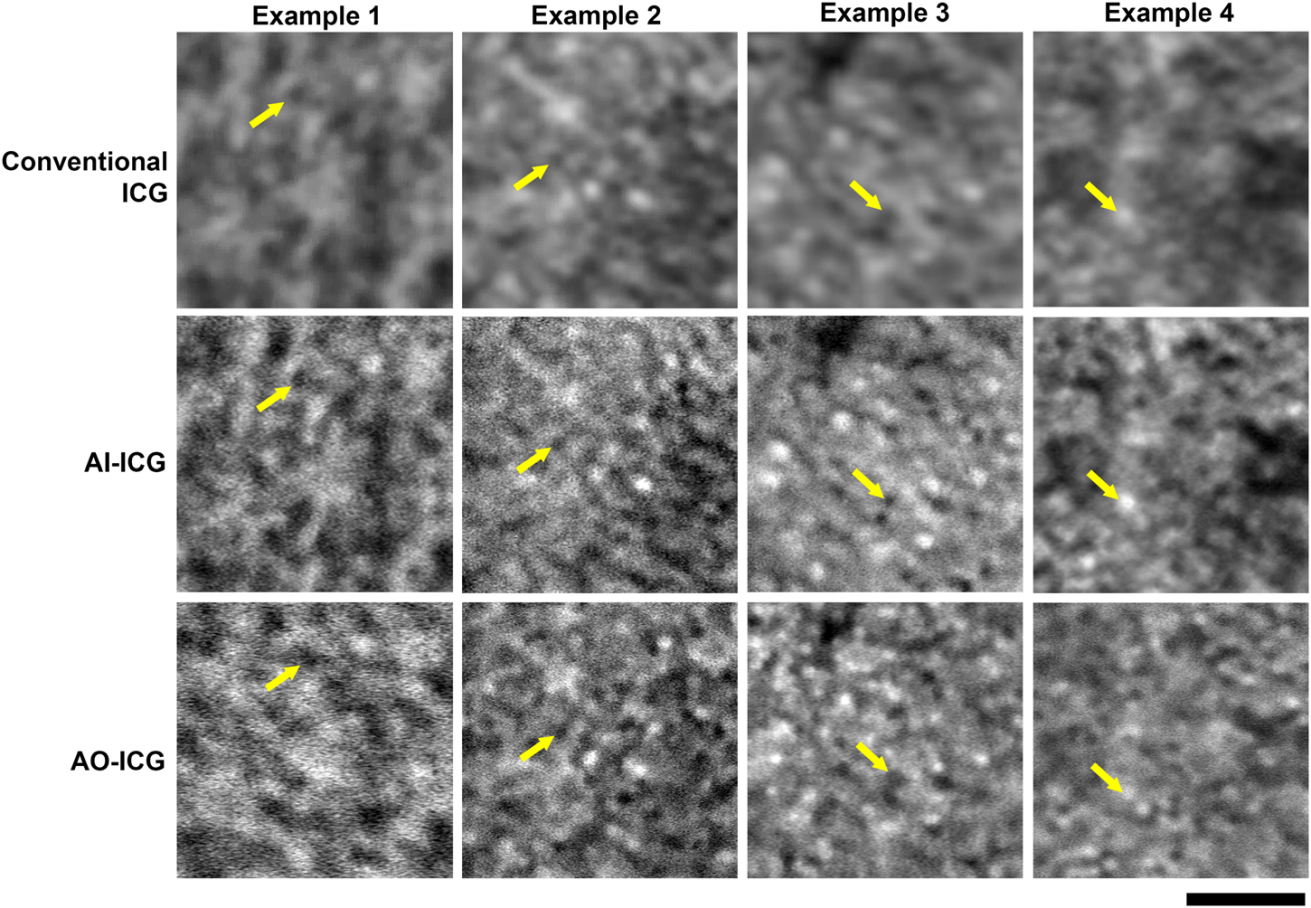
Cellular-level indocyanine green (ICG) retinal pigment epithelial (RPE) cell images from healthy eyes can be generated using stratified cycleGAN. Representative foveal RPE images from four separate eyes are shown. Conventional ICG and adaptive optics enhanced ICG (AO-ICG) images from the same location are acquired from each eye, and stratified cycleGAN image enhancement was applied to the conventional ICG image to generate the artificial intelligence assisted ICG (AI-ICG) image. Individual RPE cells can be identified (yellow arrows) in all three ICG image modalities though at different quality. Results show that AI-ICG RPE images demonstrate visibly improved quality compared to their corresponding conventional ICG images, and closely resemble the ground truth AO-ICG images. Scale bar: 100 µm.

We evaluated the performance of stratified cycleGAN on image enhancement first using a set of never-seen test images from healthy eyes (Fig. 3). Data show that AI enhancement greatly improves the visual quality of the ICG images (AI-ICG vs. conventional ICG images). These results suggest that the AI-ICG approach is capable of synthesizing images with improved resolution and contrast with close resemblance to ground truth AO-ICG images acquired at the same retinal locations for the purposes of comparison (these AO-ICG images were not provided to the stratified cycleGAN). These qualitative observations were corroborated by quantification of precision, recall, and F1-score using objective cell detection (Supplementary Table 4). In conventional ICG images, individual cells are more difficult to distinguish due to the lower resolution and contrast, but were better visualized in the enhanced AI-ICG images, establishing the ability of AI to draw out existing information content into a manner that is more accessible for routine clinical use.

**Fig. 4:**
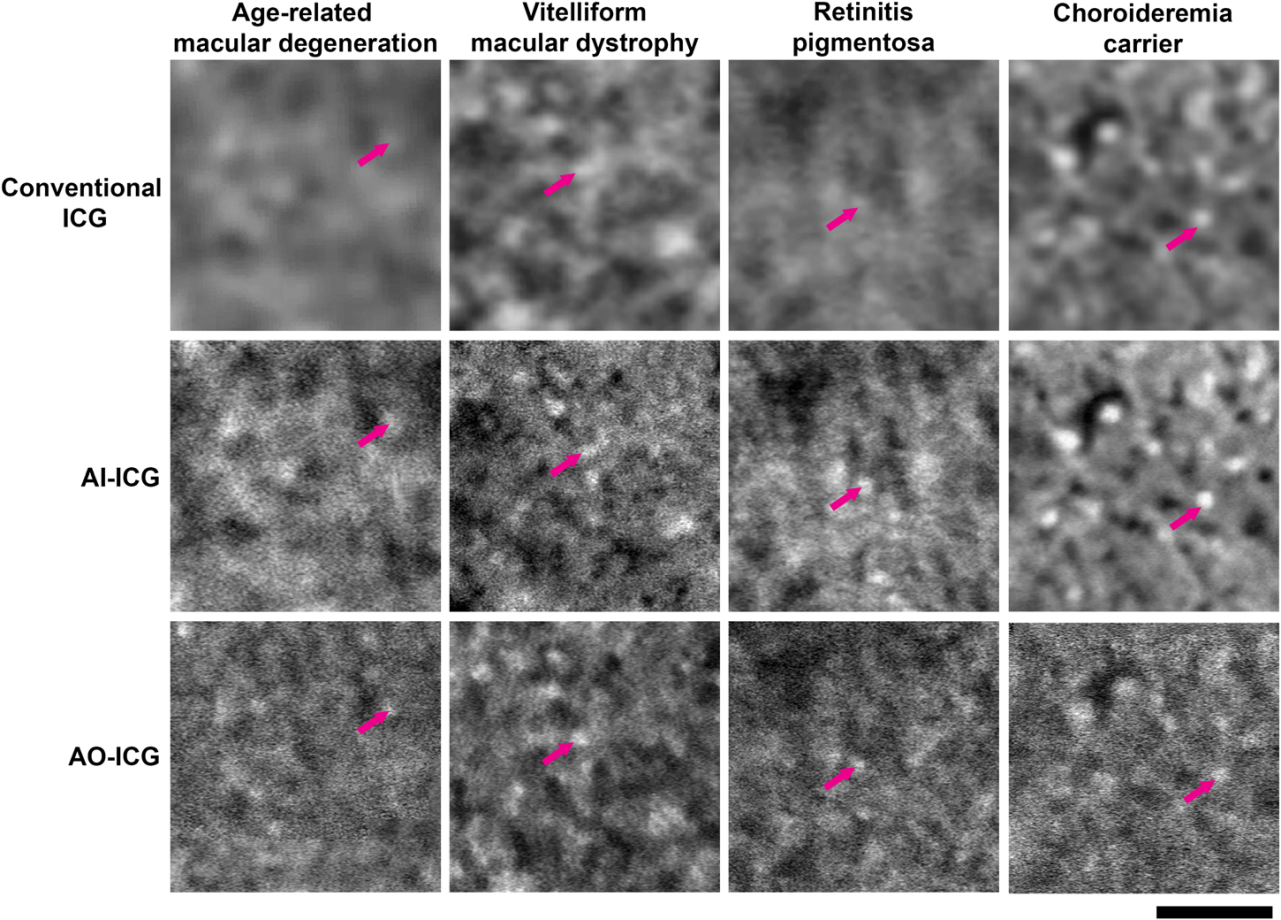
Cellular-level indocyanine green (ICG) retinal pigment epithelial (RPE) cell images from diseased eyes generated using stratified cycleGAN. Late phase ICG images from four different diseases (before and after artificial intelligence (AI) enhancement) are shown. Images shown were acquired from retinal locations ~0.5 mm away from the fovea for age-related macular degeneration (AMD), retinitis pigmentosa, and choroideremia carrier, and 3 mm for vitelliform macular dystrophy in an area with no apparent outer retinal lesions. Conventional ICG and ground truth adaptive optics (AO)-ICG images were acquired from identical locations. Stratified cycleGAN trained on healthy data only was applied to the conventional ICG image to generate the AI assisted (AI-ICG) images. Examples of individual RPE cells are shown by magenta arrows. Scale bar: 100 µm.

In addition to enhancing images from healthy eyes (Fig. 3), we also demonstrated the applicability of the AI-ICG approach to enhance conventional ICG images from eyes with AMD, vitelliform macular dystrophy, retinitis pigmentosa, and a carrier of choroideremia (Fig. 4 and Supplementary Table 2). Stratified cycleGAN was applied to the images from the various diseased eyes. Despite the many differences in pathology between these four diseases, RPE cells were successfully enhanced using the AI-ICG approach and were consistent with ground-truth AO-ICG images. Notably, stratified cycleGAN was trained on only healthy data. To further evaluate whether the generated AI-ICG images have sharper features than their corresponding conventional ICG images, we performed an image sharpness test (Supplementary Fig. 6), which showed that the features in AI-ICG images were improved by ~8X compared to conventional ICG images (8.3 ± 5.9 [mean ± SD], *p* = 0.0024). These preliminary results on previously-unseen data suggest the possibility of obtaining images of the RPE cellular mosaic across both health and disease.

**Fig. 5:**
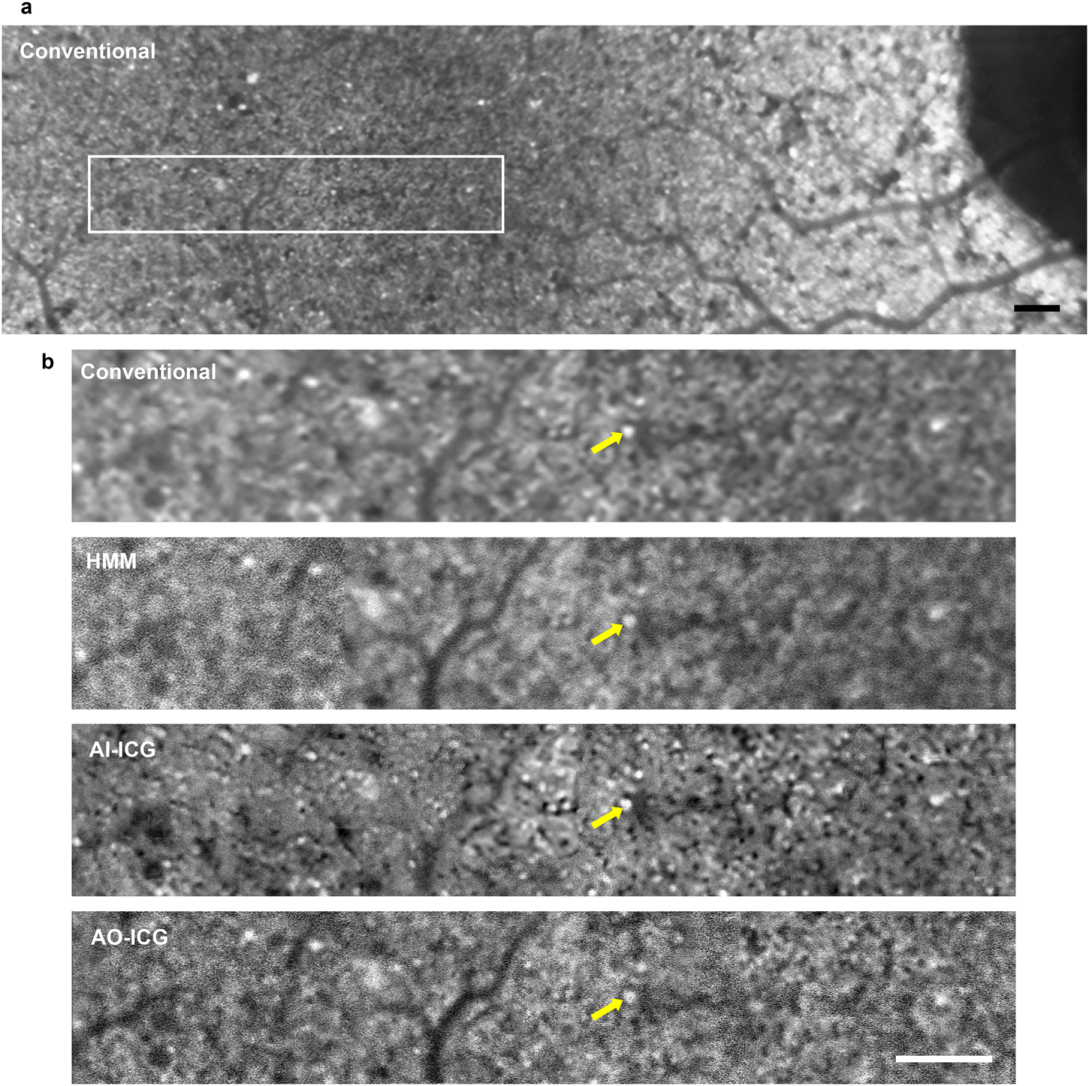
Multimodal indocyanine green (ICG) retinal pigment epithelial (RPE) cell imaging of an eye from a choroideremia carrier. **a** Conventional ICG. **b** Zoomed-in view of the ICG RPE signal (box in 5a) from conventional, high magnification module (HMM), artificial intelligence assisted (AI-ICG), and adaptive optics enhanced (AO-ICG). AI-ICG has the potential to enable large scale visualization of the RPE mosaic more efficiently than AO. An example of a single cell is shown (arrows). The contrast of HMM image was enhanced for visualization purposes only. All scale bars: 200 µm.

Finally, we used the AI-assisted imaging approach to generate a montage of the RPE mosaic in a manner that was much more efficient than would have been possible with AO imaging alone. The amount of retinal area that can be covered in one AO imaging session is typically limited to smaller fields of view (~0.5 mm by 0.5 mm) when compared to clinical instruments (>9 mm by 9 mm). Here, we show that AI-ICG can be leveraged to virtually generate a large-area montage of an eye from a female choroideremia carrier. Choroideremia is an X-linked retinal disease that has been shown to cause abnormal changes in RPE in female carriers^23,62,63^. Building upon our previous results, patterns of RPE fluorescence from a choroideremia carrier were also consistently observed across conventional, HMM, and AO-ICG (Fig. 5). The AI-ICG montage over the same retinal region shows a clear improvement over the corresponding clinical images with comparable or better visibility of individual RPE cells than AO-ICG for this eye (Fig. 5b). Notably, AI-ICG generated the larger-area montage in much shorter time than AO-ICG. To generate this montage of RPE cells covering the ~2 mm by 0.4 mm area demarcated in Fig. 5a using AO-ICG, a total of ~11 h was required, including image acquisition (one hour per eye) and post processing (>10 h). With AI-ICG, the image covering this same area was obtained with the assistance of AI within 3 min (~45 s conventional image acquisition and up to 2 min AI-assisted image enhancement), substantially faster than AO-ICG with a 220-fold improvement in time. This example demonstrates that cellular-level information can be acquired in a clinical environment without the use of AO. More importantly, the result shown here is potentially transformative as a strategy for improving the accessibility of next-generation imaging technology through the use of AI.

## Discussion

Building upon published findings that demonstrate AO-ICG can be used to visualize and track the in situ mosaicism of RPE at the foveal center in living human eyes^19^, this study gathered AO-ICG data from 26 healthy eyes across a broad age range (22–63 years old) and across a range of retinal locations to evaluate and compare in vivo RPE parameters acquired from AO-ICG to published values. Results show that both the RPE cell-to-cell spacing and density measurements based on AO-ICG images are consistent with the values previously published by other imaging studies and histology, and the highest RPE density is observed in the fovea as previously described (Fig. 1b)^18,19,44–57,59^. In addition, our results demonstrate an in vivo relationship between RPE packing and age that expands upon published literature (Fig. 1c)^45,47,48,58,59,64^. To our knowledge, we present the largest in vivo normative dataset for RPE cell structure in living human eyes, which will be particularly important for future studies using both AO and non-AO imaging to assess the RPE.

Since AO imaging is currently not as widely accessible in routine clinical practice, and because tracking the health of RPE is an important step in understanding disease progression, extension of this technique into non-AO instrumentation is crucial. The introduction of HMM by Heidelberg Engineering enabled higher resolution imaging of structures resembling photoreceptors^61,65,66^, but to our knowledge, has never been extended to fluorescent imaging before. Compared to photoreceptors, RPE cells are larger and therefore potentially easier to resolve with non-AO instrumentation^23^. The striking similarity in ICG fluorescence patterns observed across AO, conventional, and HMM (Fig. 2b and Supplementary Videos 1 and 2) not only serve to cross-validate the source of the ICG signal being imaged^19^, but also, opens up the possibility of using an existing module (HMM) in a novel manner to visualize RPE cells in living eyes without AO. It is important however to acknowledge that the use of the HMM module has inherent challenges which can affect image quality, such as motion artifacts or uncorrected optical aberrations^61,66^. Similar to other studies, skilled and experienced operators are needed for HMM use^61^. All in all, our results clearly show that the heterogeneous pattern of ICG fluorescence observed in the RPE is not a phenomenon exclusive to AO imaging, but rather, a physiologically robust event following ICG injection that can be seen using standard instruments.

Recognizing that there is a true cellular signal on the non-AO images, in order to further improve the visualization of RPE on clinical images, we introduced an AI-based approach to enhance the appearance of RPE cells so that they resemble the quality of AO images (Fig. 3). Although training data in diseased eyes was not available, our preliminary tests on several different diseases demonstrate the potential for this model to be expanded for other purposes as more training data becomes available (Figs. 4 and 5). The proposed AI-based approach not only offers a substantial time savings (220-fold improvement from 11 h to 3 min), but also, minimal operator training is required for obtaining AI-ICG images. This strategy also illustrates the possibility of using AI as an add-on module to existing clinical instrumentation, which has the potential for revolutionizing the way in which diseases are detected and diagnosed. In the future, since the training mechanism of stratified cycleGAN only involves pairs of “low quality” images and “high quality” images, this type of enhancement training platform can be applied to different imaging modalities and disease conditions by further increasing the amount of training data. The generalizability of this image enhancement technique can potentially be redesigned to provide clinicians additional cellular information through other types of clinical imaging instruments.

Recently, AI has demonstrated promising diagnostic potential in the field of ophthalmology, including detecting diabetic retinopathy and predicting the progression of AMD^2,3^. In this study, we further extend the range of applications of AI in ophthalmology. Considering that the RPE shows age-dependent changes and plays a critical role in maintaining eye health, routine visualization and assessment of the RPE at the cellular level is critical for understanding age-related eye diseases. To date, ICG imaging has mostly been used for visualizing the choroid and choroidal circulation. The data presented in this study demonstrates an application of ICG beyond angiography. By incorporating AI-assisted image enhancement with ICG imaging, it is now possible to obtain cellular information of RPE in addition to choroidal vascularization using the same dye in the clinic. More broadly, visualizing the status of cells directly in the living human eye could enable new discoveries for detecting and monitoring the initiation and progression of neurodegenerative disease^67^. With more routine access to cellular scale information in the clinic, it may be possible to better detect the onset of diseases as well as monitor cellular changes in response to novel treatments in upcoming clinical trials.

## Supplementary information

Supplementary Information File

Supplementary Video 1

Supplementary Video 2

Supplementary Data

Reporting Summary

Description of Additional Supplementary Materials

## Data Availability

The data supporting the findings of this study are available within the paper and its Supplementary Information files. The source data for Figs. 1 and 2, and Supplementary fig. 4 and 6 can be found in the Supplementary Data file.

## Code availability

The implementation of stratified cycleGAN is publicly available in the Zenodo repository (10.5281/zenodo.14866971)^68^.
