# Supplementary material for: Artificial intelligence assisted clinical fluorescence imaging achieves in vivo cellular resolution comparable to adaptive optics ophthalmoscopy
Source: Commun Med (Lond). 2025 Apr 23;5:105. doi: 10.1038/s43856-025-00803-z (PMC12019174; doi:10.1038/s43856-025-00803-z)
Supplement: Supplementary file 1 — Supplementary Information File [file 43856_2025_803_MOESM1_ESM.pdf]

Supplementary Information

Artificial intelligence assisted clinical fluorescence imaging achieves in vivo cellular resolution comparable to adaptive optics ophthalmoscopy

Joanne Li, Jianfei Liu, Vineeta Das, Hong Le, Nancy Aguilera, Andrew J. Bower, John P. Giannini, Rongwen Lu, Sarah Abouassali, Emily Y. Chew, Brian P. Brooks, Wadih M. Zein, Laryssa A. Huryn, Andrei Volkov, Tao Liu, and Johnny Tam

National Eye Institute, National Institutes of Health, Bethesda, MD 20892, USA

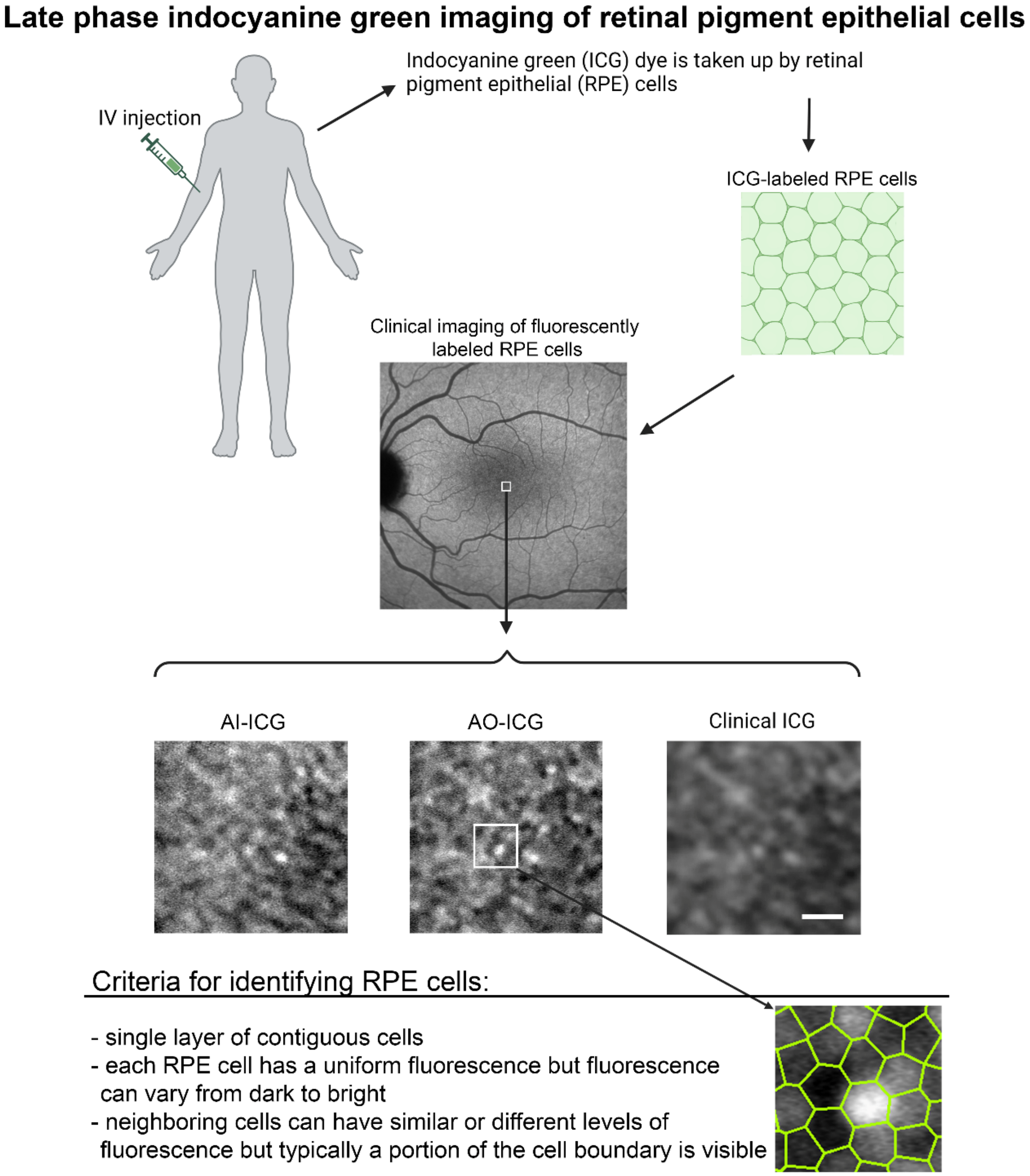

Supplementary Figure 1. Overview of late phase indocyanine green (ICG) imaging approach for visualizing retinal pigment epithelial (RPE) cells. Following intravenous (IV) injection of ICG dye, RPE cells are labeled by ICG and can be imaged using clinical instrumentation (Spectralis, Heidelberg Engineering) as well as adaptive optics enhanced ICG (AO-ICG)[1] and artificial intelligence assisted ICG (AI-ICG) imaging. The criteria for identifying individual RPE cells from late phase ICG imaging is summarized. Individual cells have uniform fluorescence, but neighboring cells can have similar or different levels of fluorescence. For visualization purposes, a Voronoi map that shows approximate outlines of RPE cells based on identifications is shown. Scale bar: 50 µm.

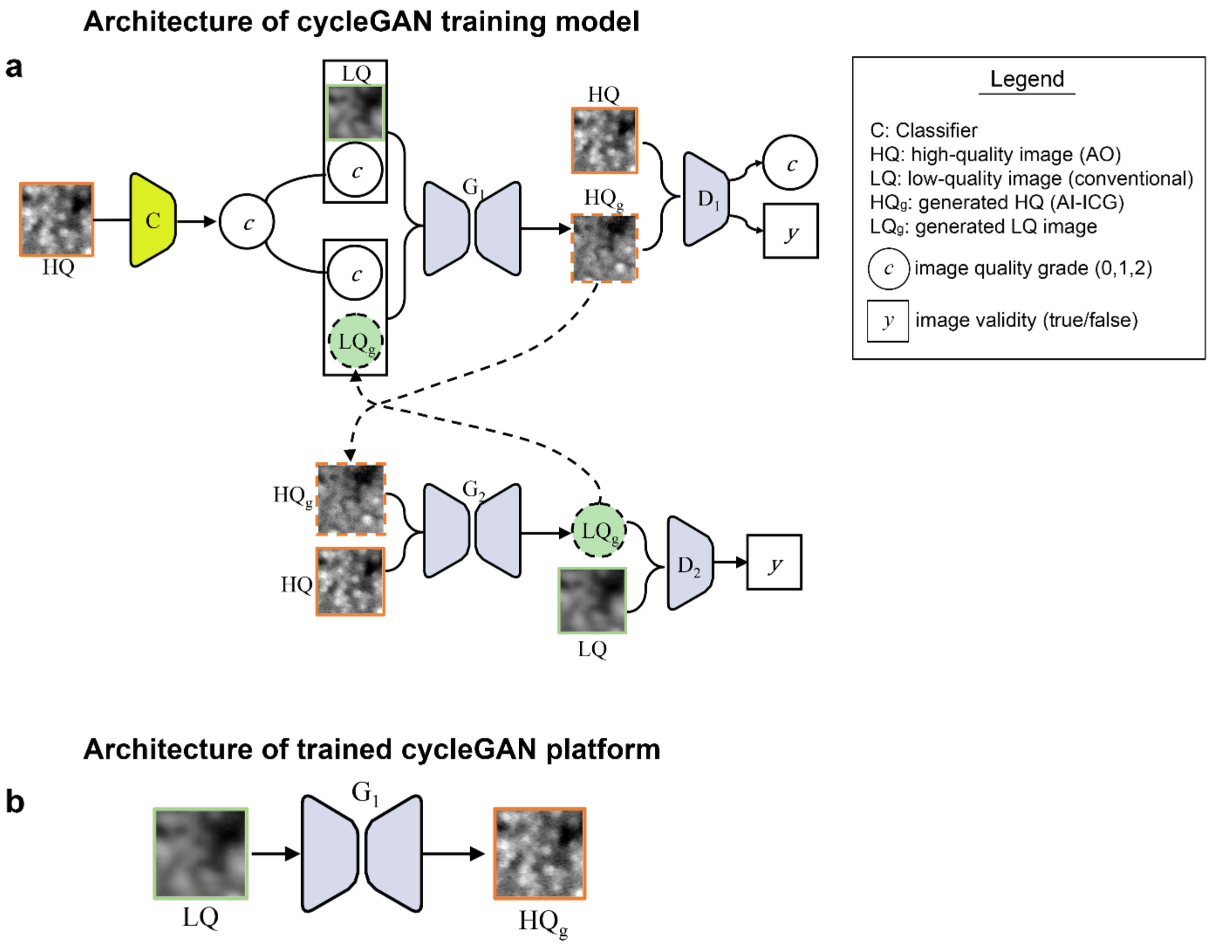

Supplementary Figure 2. Overview of stratified cycleGAN to generate artificial intelligence assisted indocyanine green (AI-ICG) images and the architecture of the training model[2]. **(a)** The architecture of the training model is shown here. A numerical image quality grade (c) is first assigned to the high quality HQ (AO) images by a classifier (C) using semi-supervised pseudo-labeling. This is combined with its corresponding low quality LQ (conventional) image and sent to a generator (G_1) to generate HQ images (HQ_g, AI-ICG). AI-ICG images are examined by a discriminator (D_1) by comparing them against the HQ images and providing the image validity (y) and quality (c) results. The quality of the generated HQ images is also checked by a second generator-discriminator (G_2-D_2) system, which produces generated LQ (LQ_g) images and compares them against the original LQ conventional images. This iterative training process continues until the discriminator cannot distinguish between the generated HQ_g image (AI-ICG) and the ground truth HQ image (adaptive optics enhanced ICG, AO-ICG). When the training is complete, generator G_1 is used for the validation and testing of the model. **(b)** After training, stratified cycleGAN takes a LQ (conventional) image and sends it to a generator (G_1) to generate a HQ_g image (AI-ICG).

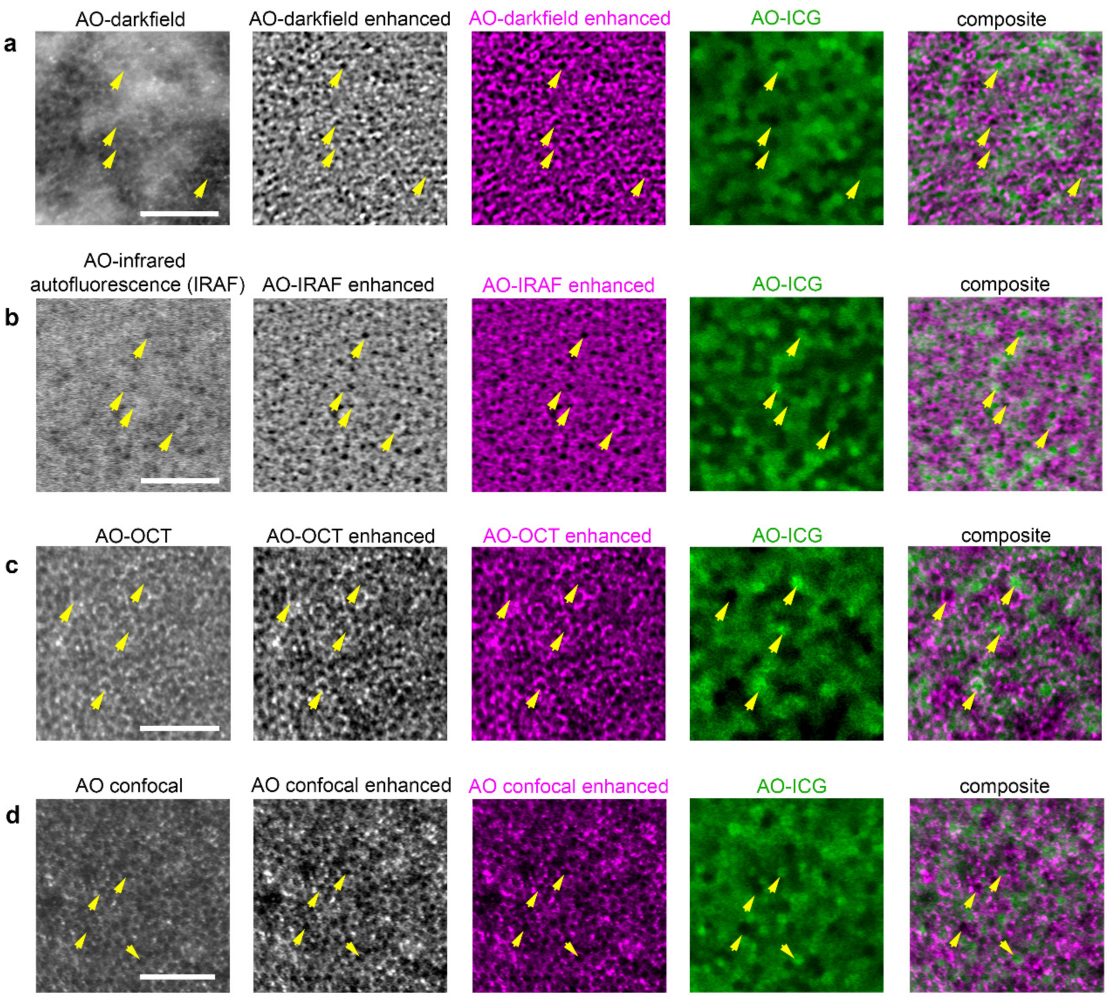

Supplementary Figure 3. Comparison of retinal pigment epithelial (RPE) cells imaged using different modalities. **(a)** Spatially co-registered adaptive optics (AO)-darkfield and AO enhanced indocyanine green (AO-ICG). In AO-ICG images the entire cell (center and surround) is labeled, whereas in AO darkfield images only the cell surrounds are shown (border plus a portion of the cell interior). For visualization purposes, a bandpass filter was applied (AO-darkfield enhanced). A color merged composite image is shown on the right. **(b)** Co-registered AO infrared autofluorescence (IRAF) and AO-ICG images. The AO-IRAF images also show cell surrounds, which were also enhanced with a bandpass filter for visualization purposes. **(c)** Co-registered AO optical coherence tomography (AO-OCT) and AO-ICG images. The AO-OCT image also shows cell surrounds and contrast was enhanced for visualization purposes. **(d)** Co-registered AO confocal reflectance and AO-ICG images from an eye in which the overlying photoreceptors have been lost, revealing the RPE mosaic[1]. In all cases, the cell surrounds colocalize with ICG-labeled RPE cells. Examples of individual RPE cells are shown in the yellow arrows. Scale bars: 100 μm.

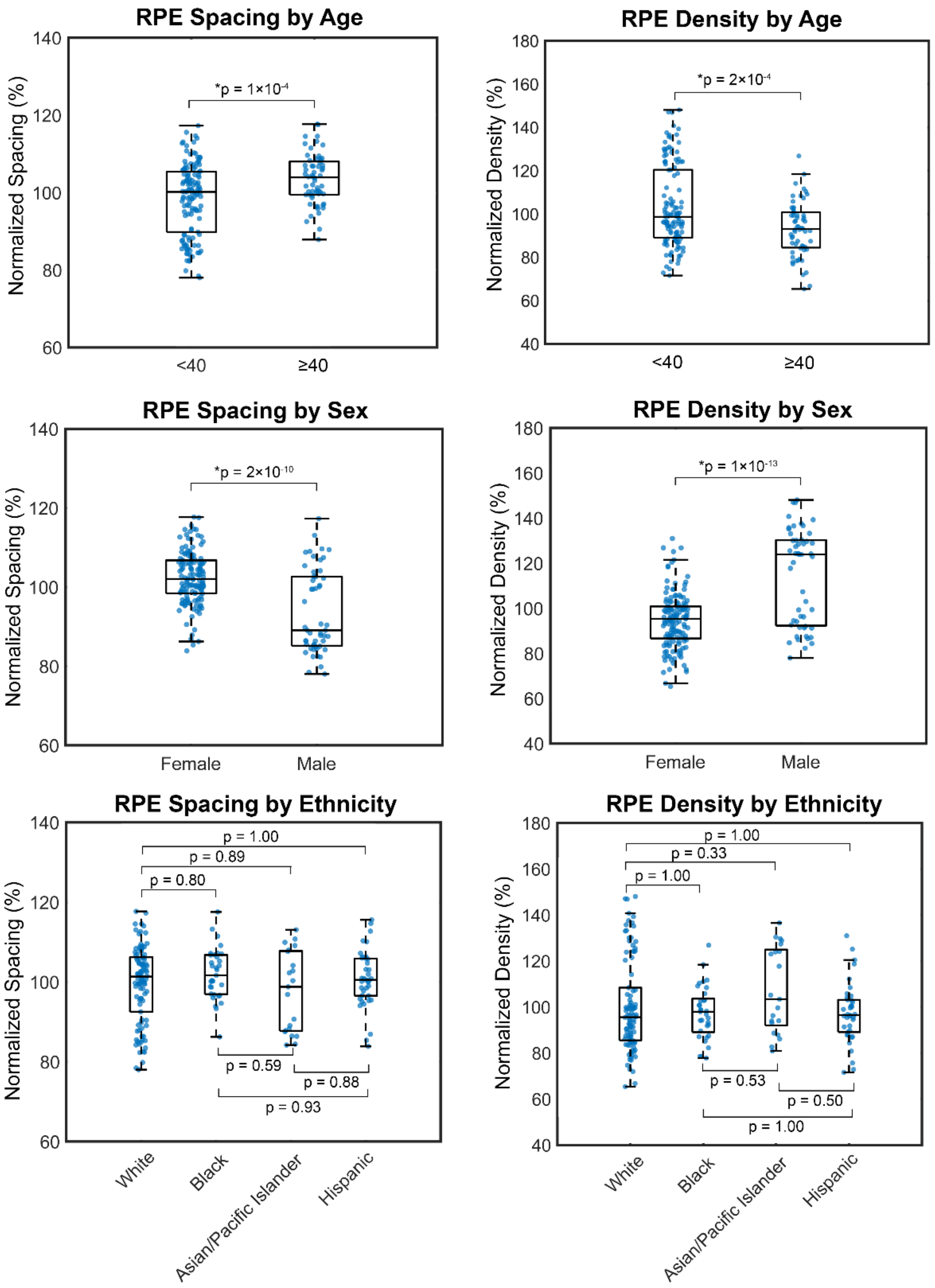

Supplementary Figure 4. Comparison of retinal pigment epithelial (RPE) cell spacing and density across age, sex, and ethnicity. The normalized spacing/density, obtained by dividing individual measurements by the expected normative values at corresponding eccentricities, was categorized by sex and ethnicity. The box plots represent the summary of each dataset (minimum, first quartile, median, third quartile, and maximum), and the data points represent the individual measurements. Statistical analysis of normalized spacing/density with age and sex was calculated using two-tailed t-test, and for ethnicity using Kruskal-Wallis paired with Tukey's honest significant difference test.

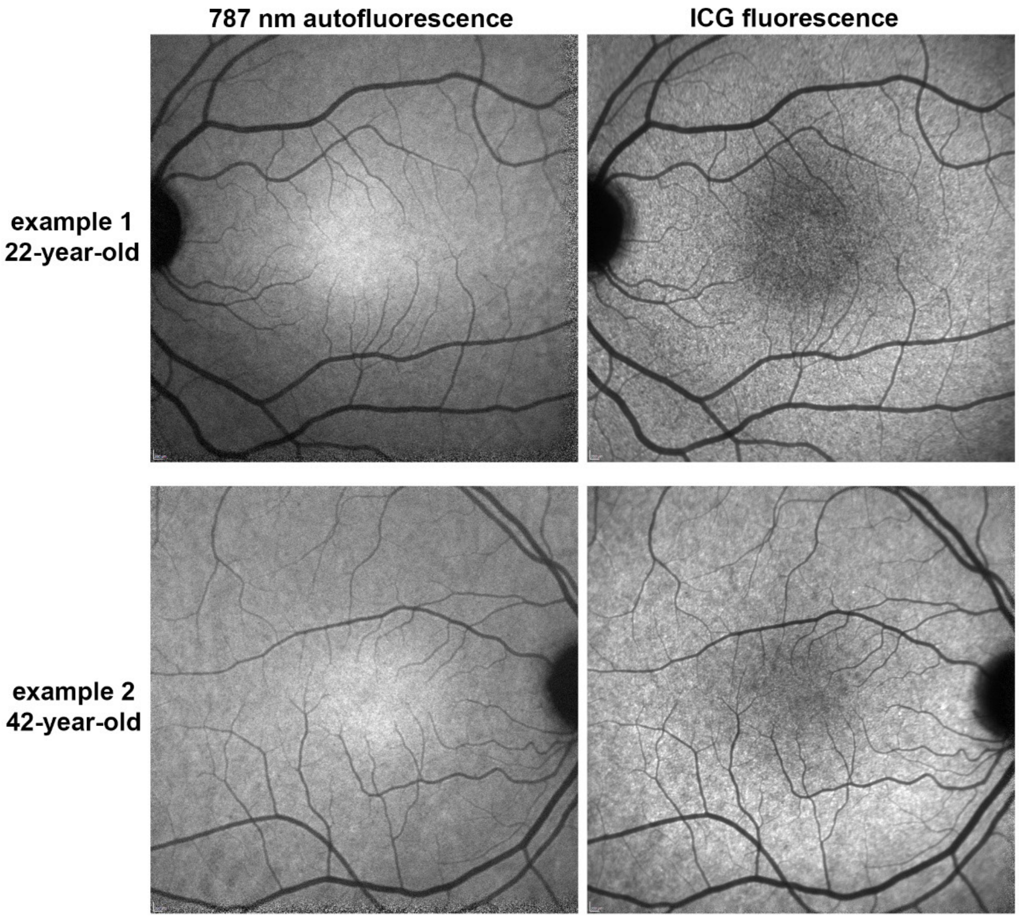

Supplementary Figure 5. Example 787-nm infrared autofluorescence (IRAF) and indocyanine green (ICG) fluorescence images from two eyes (left eye for 22-year-old and right eye for 42-year old). IRAF images show a slight increase in autofluorescence in the macula (center of the image). Late phase ICG images show a slight decrease in fluorescence (cyanescence) in the macula.

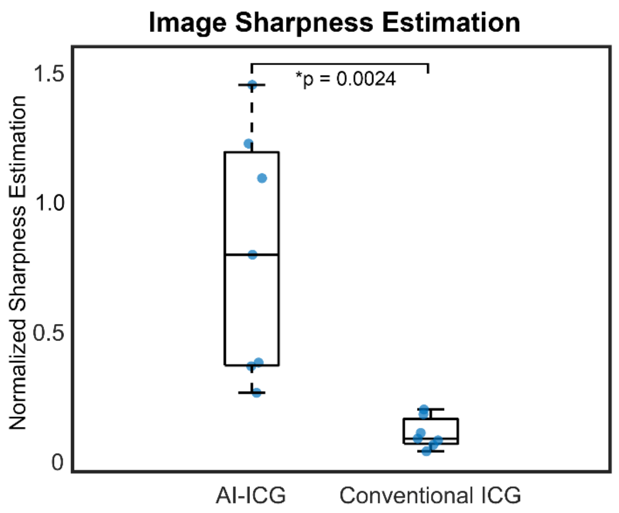

Supplementary Figure 6. Image sharpness is improved in artificial intelligence assisted indocyanine green (AI-ICG) images. A normalized comparison of the sharpness of the image features is performed on several pairs of AI-generated AI-ICG images and conventional ICG. Image sharpness was estimated using the gradient magnitude, calculated by diving the sum of all gradient norms by the number of available pixels. Using the sharpness value of adaptive optics enhanced ICG (AO-ICG) as the reference (normalized sharpness estimation for AO-ICG = 1), the sharpness value of AI-ICG and conventional ICG was then normalized by dividing by the sharpness value of AO-ICG (reference). Results show that AI-enhanced ICG images (AI-ICG) demonstrated significant improvement (8.3±5.9 times improvement, mean±SD) in image feature sharpness compared to their corresponding conventional ICG images (p=0.0024).

Supplementary Table 1. Description of healthy subject cohort

Subject	Age	Sex	Ethnicity	Eyes	Imaging Modalities
1	20-24	F	Asian/Pacific Islander	OS	Conventional, AO
2	20-24	F	Hispanic	OD	Conventional, AO, HMM
3	20-24	F	White	OS	Conventional, AO, HMM
4	20-24	F	Hispanic	OD	Conventional, AO
5	20-24	M	White	OU	Conventional, AO
6	20-24	M	White	OD	Conventional, AO
7	20-24	M	White	OD	Conventional, AO, HMM
8	20-24	F	White	OD	Conventional, AO, HMM
9	20-24	M	Black	OD	Conventional, AO
10	25-29	F	Black	OS	Conventional, AO
11	25-29	F	White	OS	Conventional, AO
12	25-29	M	White	OS	Conventional, AO
13	25-29	M	Asian/Pacific Islander	OU	Conventional, AO
14	25-29	F	White	OU	Conventional, AO
15	30-34	M	White	OS	Conventional, AO
16	35-39	F	Hispanic	OU	Conventional, AO
17	40-44	F	White	OD	Conventional, AO, HMM
18	45-49	F	White	OS	Conventional, AO, HMM
19	50-54	F	Black	OU	Conventional, AO
20	55-59	F	White	OU	Conventional, AO
21	55-59	F	White	OS	Conventional, AO
22	60-64	M	White	OS	Conventional, AO

1. OD: right eye; OS: left eye; OU: both eyes
2. AO: adaptive optics; HMM: high magnification module

Supplementary Table 2. Description of disease eyes

Subject	Age	Sex	Disease	Eye	Visual acuity	Ethnicity
1	60-64	F	Age-related macular degeneration	OS	20/20	Asian/Pacific Islander
2	20-24	M	Vitelliform macular dystrophy	OD	20/40	White
3	55-59	F	Retinitis pigmentosa	OS	20/16	Black
4	50-54	F	Choroideremia carrier	OD	20/16	White

OD: right eye; OS: left eye

Supplementary Table 3. Overview of conventional, high magnification module (HMM), and adaptive optics (AO) imaging modes

Instrument	Model	FOV (degrees)	FOV (mm)	FOV (pixels)	Wavelength (nm)
Conventional	Spectralis HRA	30°	9	~1536	788
HMM	Spectralis HRA<br>High Magnification Module	8°	2.4	~1536	788
AO	Custom AO-ICG[1]	1-2°	0.3-0.6	~605	790

- Spectralis HRA: Spectralis high resolution angiography (Heidelberg Engineering)
- ICG: indocyanine green
- FOV: square field-of-view
- Both conventional and HMM imaging are considered to be non-AO.
- Wavelength refers to excitation wavelength.

Supplementary Table 4. Performance of stratified cycleGAN based on objective cell detection

Precision (mean±SD%)	Recall (mean±SD%)	F1-score (mean±SD%)
80.8±7.6	90.9±6.5	85.0±3.4

The performance of the model was evaluated based on objective cell detection accuracy. Retinal pigment epithelial (RPE) cell detection was performed on artificial intelligence (AI)-generated indocyanine green (ICG) RPE images (AI-ICG) as well as on their corresponding ground truth adaptive optics (AO)-ICG images using an AI-trained RPE cell detection software. The performance of stratified cycleGAN on image enhancement was then evaluated by calculating the precision, recall, and F1 score of the RPE detection result on AI-ICG compared to that on AO-ICG. Precision score calculates the proportion of detected cells that are correct, recall the fraction of cells that was identified, and F1 score the harmonic mean of the precision and recall. Higher scores indicate better cell detection accuracy.

Supplementary Information References:

1. Jung, H., Liu, T., Liu, J., Huryn, L. A. & Tam, J. Combining multimodal adaptive optics imaging and angiography improves visualization of human eyes with cellular-level resolution. *Commun. Biol.* **1**, 189 (2018). https://doi.org:10.1038/s42003-018-0190-8
2. Liu, J., Li, J., Liu, T. & Tam, J. Graded image generation using stratified CycleGAN. In *Medical Image Computing and Computer Assisted Intervention – MICCAI 2020.* 760-769 (Springer International Publishing).
