# Supplementary material for: Artificial intelligence assisted clinical fluorescence imaging achieves in vivo cellular resolution comparable to adaptive optics ophthalmoscopy
Source: Commun Med (Lond). 2025 Apr 23;5:105. doi: 10.1038/s43856-025-00803-z (PMC12019174; doi:10.1038/s43856-025-00803-z)
Supplement: Supplementary file 5 — Reporting Summary [file 43856_2025_803_MOESM5_ESM.pdf]

nature portfolio

Corresponding author(s): Johnny Tam

Last updated by author(s): Feb 14, 2025

Reporting Summary

Nature Portfolio wishes to improve the reproducibility of the work that we publish. This form provides structure for consistency and transparency in reporting. For further information on Nature Portfolio policies, see our Editorial Policies and the Editorial Policy Checklist.

Statistics

For all statistical analyses, confirm that the following items are present in the figure legend, table legend, main text, or Methods section.

n/a	Confirmed	
☐	☒	The exact sample size (*n*) for each experimental group/condition, given as a discrete number and unit of measurement
☐	☒	A statement on whether measurements were taken from distinct samples or whether the same sample was measured repeatedly
☐	☒	The statistical test(s) used AND whether they are one- or two-sided<br>*Only common tests should be described solely by name; describe more complex techniques in the Methods section.*
☒	☐	A description of all covariates tested
☒	☐	A description of any assumptions or corrections, such as tests of normality and adjustment for multiple comparisons
☐	☒	A full description of the statistical parameters including central tendency (e.g. means) or other basic estimates (e.g. regression coefficient) AND variation (e.g. standard deviation) or associated estimates of uncertainty (e.g. confidence intervals)
☒	☐	For null hypothesis testing, the test statistic (e.g. *F*, *t*, *r*) with confidence intervals, effect sizes, degrees of freedom and *P* value noted<br>*Give P values as exact values whenever suitable.*
☒	☐	For Bayesian analysis, information on the choice of priors and Markov chain Monte Carlo settings
☒	☐	For hierarchical and complex designs, identification of the appropriate level for tests and full reporting of outcomes
☒	☐	Estimates of effect sizes (e.g. Cohen's *d*, Pearson's *r*), indicating how they were calculated

Our web collection on statistics for biologists contains articles on many of the points above.

Software and code

Policy information about availability of computer code

Data collection	Data collection was performed using a custom retinal imaging system that has previously been reported (ref 18)
Data analysis	Matlab, Python, Microsoft Excel, and Adobe Photoshop were used for data visualization and analysis.

For manuscripts utilizing custom algorithms or software that are central to the research but not yet described in published literature, software must be made available to editors and reviewers. We strongly encourage code deposition in a community repository (e.g. GitHub). See the Nature Portfolio guidelines for submitting code & software for further information.

Data

Policy information about availability of data

All manuscripts must include a data availability statement. This statement should provide the following information, where applicable:

- Accession codes, unique identifiers, or web links for publicly available datasets
- A description of any restrictions on data availability
- For clinical datasets or third party data, please ensure that the statement adheres to our policy

All data are available in the main text or supplementary materials.

Research involving human participants, their data, or biological material

Policy information about studies with human participants or human data. See also policy information about sex, gender (identity/presentation), and sexual orientation and race, ethnicity and racism.

Reporting on sex and gender	The study participants include unaffected subjects (33.6+/-13.8 years old, 14 females and 6 males), and 4 affected patients with 3 females (63, 59, and 51 years old) and 1 male (22 years old).
Reporting on race, ethnicity, or other socially relevant groupings	The study participants (both unaffected and affected) include 15 Whites, 3 Blacks, 3 Asian/Pacific Islanders, and 3 Hispanics.
Population characteristics	The participants include both subjects with no signs of ocular diseases and those with retinal diseases.
Recruitment	Subjects were recruited from the National Eye Institute Eye Clinic at the National Institutes of Health in Bethesda, Maryland. Informed consent was obtained from all participants prior to enrollment. All participants received a comprehensive dilated eye exam.
Ethics oversight	This study was approved by the institute review board of the National Institutes of Health and was conducted in accordance with the Declaration of Helsinki.

Note that full information on the approval of the study protocol must also be provided in the manuscript.

Field-specific reporting

Please select the one below that is the best fit for your research. If you are not sure, read the appropriate sections before making your selection.

☒ Life sciences ☐ Behavioural & social sciences ☐ Ecological, evolutionary & environmental sciences

For a reference copy of the document with all sections, see nature.com/documents/nr-reporting-summary-flat.pdf

Life sciences study design

All studies must disclose on these points even when the disclosure is negative.

Sample size	Expanding upon previous reports (refs 18, 19), normative data were constructed from 26 healthy eyes from 20 participants between 22 and 63 years old. In addition, images from 4 disease eyes from 4 affected patients were included to demonstrate the potential of AI-assisted image enhancement. The number of participants included in this study was larger than the typical sample sizes for adaptive optics indocyanine green imaging studies.
Data exclusions	Since adaptive optics video sequences were captured, we excluded any raw data captured during blinks, excessive eye motion, or other drop in image quality that prevented image sequences from being registered to a reference frame, which is the standard approach for adaptive optics retinal imaging data.
Replication	Adaptive optics and conventional retinal imaging were performed on 24 subjects of varied age groups at different retinal locations. The designed method was successful in visualizing retinal pigment epithelial cells in all participants using indocyanine green (ICG) fluorescent dye. In addition, AI-assisted image enhancement was applied on conventional ICG images, which successfully enhanced the image quality of conventional images of adaptive optics-like cellular-resolution images.
Randomization	Randomization was not applicable. Participants were categorized based on outcomes of their eye examinations (healthy or disease) and their age, sex, and ethnicity.
Blinding	Blinding was not applicable since no anticipated result was expected.

Reporting for specific materials, systems and methods

We require information from authors about some types of materials, experimental systems and methods used in many studies. Here, indicate whether each material, system or method listed is relevant to your study. If you are not sure if a list item applies to your research, read the appropriate section before selecting a response.

Materials & experimental systems

n/a	Involved in the study
☒	☐ Antibodies
☒	☐ Eukaryotic cell lines
☒	☐ Palaeontology and archaeology
☒	☐ Animals and other organisms
☐	☒ Clinical data
☒	☐ Dual use research of concern
☒	☐ Plants

Methods

n/a	Involved in the study
☒	☐ ChIP-seq
☒	☐ Flow cytometry
☒	☐ MRI-based neuroimaging

Clinical data

Policy information about clinical studies

All manuscripts should comply with the ICMJE guidelines for publication of clinical research and a completed CONSORT checklist must be included with all submissions.

Clinical trial registration: NCT02317328

Study protocol: Adaptive Optics Retinal Imaging

Data collection: All data were collected at the National Eye Institute Eye Clinic at the National Institutes of Health in Bethesda, Maryland.

Outcomes: The primary outcome is qualitative and quantitative assessment of AO images from healthy and disease eyes.

Plants

Seed stocks: n/a

Novel plant genotypes: n/a

Authentication: n/a
