# Supplementary material for: Artificial intelligence assisted clinical fluorescence imaging achieves in vivo cellular resolution comparable to adaptive optics ophthalmoscopy
Source: Commun Med (Lond). 2025 Apr 23;5:105. doi: 10.1038/s43856-025-00803-z (PMC12019174; doi:10.1038/s43856-025-00803-z)
Supplement: Supplementary file 6 — Description of Additional Supplementary Materials [file 43856_2025_803_MOESM6_ESM.docx]

**Description of Additional Supplementary Files**

**File name:** Supplementary Data

**Description:** The source data for Figures 1 and 2, and Supplementary Figures 4 and 6

**File name:** Supplementary Video 1

**Description:** Spatially-registered conventional, HMM, and AO-ICG montage of the macula from a healthy eye. The contrast of the non-AO images was adjusted for visualization purposes. Individual RPE cells can be identified as a mosaic of bright and dark cells. Neighboring cells can have different or similar level of ICG intensity. A consistent pattern of fluorescence is observed across all imaging modes, suggesting the late-phase ICG pattern seen in RPE cells can be visualized by both AO and non-AO imaging. Scale bar: 200 µm.

**File name:** Supplementary Video 2

**Description:** A zoom-in view of the spatially-registered conventional, HMM, and AO-ICG images from the macula of a healthy eye. In this smaller field-of-view, details of the ICG mosaic across the three imaging modes can be better compared, in which AO-ICG demonstrates the highest resolution, followed by HMM and the conventional ICG.
